# Supplementary material for: Labor unionization and real earnings management: Evidence from labor elections
Source: PLoS One. 2024 Feb 20;19(2):e0292889. doi: 10.1371/journal.pone.0292889 (PMC10878506; doi:10.1371/journal.pone.0292889)
Supplement: S1 Appendix — (DOCX) [file pone.0292889.s001.docx]

# APPENDIX TABLE A1: Definitions and Data Sources of Main Variables

| **Variable** | **Definition** | **Source** |
| --- | --- | --- |
|  | | |
| *REM1* | Based on [20], the measure of REM is the negative sum of abnormal operating cash flow and abnormal discretionary expenses:  *REM= − REM_OANCF-REM_DISX*  where *REM_OANCF* denotes abnormal operating cash flow and *REM_DISX* denotes abnormal discretionary expenses. | COMPUSTAT |
| *REM2* | Based on [62], the measure of REM is the abnormal production costs minus abnormal discretionary expenses:  *REM1=REM_PROD − REM_DISX*  where *REM_PROD* denotes abnormal production costs and *REM_DISX* denotes abnormal discretionary expenses. | COMPUSTAT |
| *REM1_R* | *REM_OANCFR* is used instead of *REM_OANCF* to calculate *REM*, where *REM_OANCFR* is defined by [40]:  $\frac{OANCF_{it}}{AT_{i,t-1}}=\alpha_{0}+\alpha_{1}\frac{SALE_{it}}{AT_{i,t-1}}+\alpha_{2}\frac{\Delta SALE_{it}}{AT_{i,t-1}}+\alpha_{3}\frac{PROD_{it}}{AT_{i,t-1}}+\alpha_{4}\frac{{DISX}_{it}}{AT_{i,t-1}}+\varepsilon_{it}$ |  |
| *REM_DISX* | Based on [60], abnormal production costs are the residual of the following equation:  $\frac{DISX_{it}}{AT_{i,t-1}}=\alpha_{0}+\alpha_{1}\frac{1}{AT_{i,t-1}}+\alpha_{2}\frac{SALE_{it}}{AT_{i,t-1}}+\varepsilon_{it}$  where *DISX* is the sum of advertising, R&D, and SG&A expenses. The regression runs for each industry and year with more than 15 observations. The industry is classified according to SIC two-digit codes. | COMPUSTAT |
| *REM_OANCF* | Based on [60], abnormal operating cash flow is the residual of the following equation:  $\frac{OANCF_{it}}{AT_{i,t-1}}=\alpha_{0}+\alpha_{1}\frac{1}{AT_{i,t-1}}+\alpha_{2}\frac{SALE_{it}}{AT_{i,t-1}}+\alpha_{3}\frac{\Delta SALE_{it}}{AT_{i,t-1}}+\varepsilon_{it}$  where *OANCF* denotes the operating cash flow. The regression runs for each industry and year with more than 15 observations. | COMPUSTAT |
| *REM_PROD* | Based on [60], abnormal discretionary expenses are defined as the residual of the following equation:  $\frac{PROD_{it}}{AT_{i,t-1}}=\alpha_{0}+\alpha_{1}\frac{1}{AT_{i,t-1}}+\alpha_{2}\frac{SALE_{it}}{AT_{i,t-1}}+\alpha_{3}\frac{\Delta SALE_{it}}{AT_{i,t-1}}+\alpha_{4}\frac{\Delta SALE_{i,t-1}}{AT_{i,t-1}}+\varepsilon_{it}$  where *PROD* is the sum of the cost of goods sold and changes in inventory during the year. The regression runs for each industry and year with more than 15 observations. | COMPUSTAT |
| *SIZE* | The logarithm of 1 plus the book value of total assets (*AT*) | COMPUSTAT |
| *TOBINQ* | The ratio of market value to book value (*AT*), where market value is defined as total assets (*AT*) minus common equity (*CEQ*) and deferred taxes (*TXDB*) plus the market equity *(PRCC_F* *× CSHO*) | COMPUSTAT |
| *LEV* | Long-term debt (*DLTT*) and debt in current liabilities (*DLC*) scaled by the book value of total assets (*AT*) | COMPUSTAT |
| *ROA* | The ratio of income before extraordinary items (*IBC*) to book value of total assets (*AT*) | COMPUSTAT |
| *PPENT* | Property, plant, and equipment (*PPENT*) scaled by the book value of total assets (*AT*) | COMPUSTAT |
|  |  |  |
| *SGR* | Sales growth rate, defined as the ratio of the change in sales (*SALE*) in the current year to sales in the previous year | COMPUSTAT |
| *VOL* | Stock return volatility: the standard deviation of monthly returns during the year | COMPUSTAT |
| *RTW* | A dummy variable indicating whether a firm was headquartered in a right-to-work state at the time when right-to-work law was enacted in the state | NRTW |
| *Benchmark* | Analysts’ earnings forecasts obtained from I/B/E/S | I/B/E/S |
| *Cashflow* | Operating cash flow (*OANCF*) scaled by the book value of total assets (*AT*) | COMPUSTAT |
| *Altman* | Altman Z-score=1.2 × (working capital ÷ total assets) + 1.4 × (retained earnings ÷ total assets) + 3.3 × (earnings before interest and tax ÷ total assets) + 0.6 × (market value of equity ÷ total liabilities) + 1.0 × (sales ÷ total assets) | COMPUSTAT |
| *DA_KOTHARI* | Discretionary accruals based on a performance-matched model ([51]) | COMPUSTAT |
| *DA_MJONES* | Discretionary accruals based on the modified Jones model ([26]) | COMPUSTAT |
| *DA_JONES* | Discretionary accruals based on the Jones model ([49]) | COMPUSTAT |
| *UNION_IND* | Total number of union members over the total number of employees in the industry | Unionstats |
| *UNION_COV_IND* | Total number of employees covered by labor unions over the total number of employees in the industry | Unionstats |
|  | | |
