# Supplementary material for: Labor unionization and real earnings management: Evidence from labor elections
Source: PLoS One. 2024 Feb 20;19(2):e0292889. doi: 10.1371/journal.pone.0292889 (PMC10878506; doi:10.1371/journal.pone.0292889)
Supplement: S2 Appendix — (DOCX) [file pone.0292889.s002.docx]

# APPENDIX TABLE A2: Summary Statistics of Accrual-based Earnings Management Measures

|  | **N** | **Mean** | **St.Dev** | **p25** | **Median** | **p75** |
| --- | --- | --- | --- | --- | --- | --- |
| *ΔDA_KOTHARI* | 654 | 0.004 | 0.081 | -0.032 | 0.000 | 0.037 |
| *ΔDA_MJONES* | 654 | 0.004 | 0.077 | -0.034 | 0.000 | 0.032 |
| *ΔDA_JONES* | 654 | 0.004 | 0.077 | -0.034 | -0.000 | 0.033 |

*Note:* This table shows the summary statistics of measures on accrual-based earnings management. Accural-based earnings management is measured by discretionary accruals based on discretionary accruals adjusted by firms’ past performance ([51]), modified Jones model ([26]), and discretionary accruals based on the Jones model ([49]), respectively.
