# Supplementary material for: Labor unionization and real earnings management: Evidence from labor elections
Source: PLoS One. 2024 Feb 20;19(2):e0292889. doi: 10.1371/journal.pone.0292889 (PMC10878506; doi:10.1371/journal.pone.0292889)
Supplement: S3 Appendix — (DOCX) [file pone.0292889.s003.docx]

# APPENDIX TABLE A3: Multivariate OLS Analysis: Summary Statistics

|  | **N** | **Mean** | **St.Dev** | **p25** | **Median** | **p75** |
| --- | --- | --- | --- | --- | --- | --- |
| *REM1* | 92,147 | 0.008 | 0.179 | -0.064 | 0.005 | 0.076 |
| *REM2* | 92,147 | 0.009 | 0.213 | -0.070 | 0.005 | 0.083 |
| *UNION_IND* | 92,147 | 0.097 | 0.076 | 0.040 | 0.071 | 0.136 |
| *UNION_COV_IND* | 92,147 | 0.106 | 0.080 | 0.046 | 0.079 | 0.146 |
| *SIZE* | 92,147 | 5.689 | 2.216 | 4.051 | 5.538 | 7.205 |
| *LEV* | 92,147 | 0.397 | 0.200 | 0.236 | 0.390 | 0.538 |
| *TOBINQ* | 92,147 | 2.016 | 1.588 | 1.082 | 1.484 | 2.294 |
| *ROA* | 92,147 | -0.021 | 0.200 | -0.033 | 0.034 | 0.076 |
| *SGR* | 92,147 | 0.184 | 0.535 | -0.024 | 0.084 | 0.241 |
| *PPENT* | 92,147 | 0.565 | 0.445 | 0.225 | 0.446 | 0.795 |
| *VOL* | 92,147 | 0.145 | 0.089 | 0.084 | 0.123 | 0.180 |

*Note:* This table reports the summary statistics of multivariate ordinary least squares. The financial data come from the COMPUSTAT database. The industry-level labor unionization data come from the Union Membership and Coverage database in Unionstats. Observations included in this analysis satisfy: (1) Book equity is positive; (2) All the variables used are available. Firms in financial (SIC code 6000-6999) and utility (SIC code 4900-4999) industries are excluded. All the continuous variables are winsorized at the 1% and 99% levels. Finally, the sample contains 90,755 observations during 1989-2021.
