# Supplementary material for: Labor unionization and real earnings management: Evidence from labor elections
Source: PLoS One. 2024 Feb 20;19(2):e0292889. doi: 10.1371/journal.pone.0292889 (PMC10878506; doi:10.1371/journal.pone.0292889)
Supplement: Attachment — Submitted filename: Response to reviewers.docx [file pone.0292889.s005.docx]

# **Labor Unionization and Real Earnings Management: Evidence from Labor Elections**

# **Authors’ Responses to the Reviewer Comments on PONE-D-23-09561**

We thank the editor and the two reviewers for their feedback, which strengthened our manuscript. We have carefully considered all comments from the two reviewers and revised the manuscript. The coauthor who created and analyzed the data was unavailable. So, we had to reconstruct the sample from scratch. The reconstruction means that we could extend the sample to 2021 (the earlier sample ended in 2018). However, some results have changed. Nevertheless, we believe that our responses have addressed all concerns of the reviewers.

We reproduce the reviewer’s comment in italics and our response in regular font.

# **Authors’ Responses to Reviewer 1’s Comments**

*The authors investigate whether labor unionization is associated with real earnings management. The authors find evidence to suggest that firms with labor unionization are more likely to engage in real earnings management. It is commended that the paper is well written with high coherence in the exposition. The research is important.*

We thank you for viewing the research as important and the paper as well written.

1. *The authors need to justify their regression discontinuity design (RDD) better*

Thank you for your comment. The revised manuscript (p. 3 and p. 12) better justifies the applicability of RDD in our empirical setting. In brief, a labor election supervised by the U.S. National Labor Relations Board (NLRB) results in a win for the proposed union if more than 50% of the employees vote in favor (read <https://hr.ucmerced.edu/employeelabor-relations/union-representation-faqs#:~:text=In%20order%20for%20the%20union%20to%20win%20the%20election%2C%20more,be%20represented%20by%20the%20union>). This sharp cutoff makes RDD valid in this empirical setting.

*and do more diagnostic tests and robustness checks*

The revised manuscript (pp. 13-17) includes diagnostic tests (Figures 2-3) and robustness checks (Figures 4-5 and Tables 3-4).

*For instance, is the fuzzy RDD or sharp RDD being used in the empirical test?*

We use *sharp* RDD because firms with over 50% votes are declared as unionized and any number <= 50% is declared as not unionized. The sharp cutoff of 50% allows us to use sharp RDD. Pages #3 and #12 of the revised manuscript provide this clarification.

*It is good to do balance checks to see whether pre-treatment covariates are balanced and smooth around the discontinuity point.*

Panel C of Table 1 of the revised manuscript reports that pre-treatment covariates are balanced and smooth around the discontinuity point.

*But the pre-treatment covariates should include common firm characteristics relating to firm size, growth, financial health, risks, performance, and governance.*

The revised manuscript controls for firm size, sales growth ratio, firm leverage, Tobin’s Q, collateral, return volatility, and ROA. We want to control for corporate governance. However, including corporate governance variables from BoardEx reduces the sample from 654 to 145 observations, which (given the number of regressors and fixed effects for industry, year, and state) lacks statistical power to allow us inference. Therefore, we could not control for governance here. The last paragraph of our manuscript explains this limitation and why it exists, and suggests future research to consider including covariates related to corporate governance. We thank you for helping us acknowledge this limitation.

*For robustness check, run a placebo RDD regression in which the dependent variable is replaced with the pre-treatment covariate, or include the pre-treatment covariates as the control variable in the RDD regression*

Panel B of Figure 5 and Table 4 report the RDD estimates using control variables as DVs. Thank you for this suggestion.

*re-do the RDD regression by randomly picking a non-discontinuity point for the forcing variable and the key independent variable; do a test for discontinuity in the density of the forcing variable.*

Panel A of Figure 5 reports the histogram. The X axis is the RDD estimates under artificial cutoffs between 0 and 1 other than 0.5, and the Y axis is the fraction of estimates at the cutoff.

2. *The magnitude of abnormal discretionary expense is linearly associated with, and proportional to, the extent of earnings being manipulated. However, the association between abnormal production costs and the manipulated amount of earnings is not linear nor proportional to one another, and the nonlinearity depends crucially on the amount of fixed overhead costs and the units of products produced in the current period. Therefore, please do not sum the abnormal production costs and abnormal discretionary expenditures for measuring real earnings management. Rather, do a common factor analysis to synthesize a* *composite measure based on the abnormal production costs and abnormal discretionary expenditures.*

We followed your advice and created a factor score of abnormal production costs and the manipulated amount of earnings (we name this factor variable *REM2_factor*). However, we find that *REM2_factor* and *REM2* have a correlation coefficient of 0.98. Unsurprisingly then, the regression results for *REM2* and *REM2_factor* are very similar (see the table below). Besides, Cheng, Lee, and Shevlin (2016) suggested that individual measures are better at capturing different types of real earnings management than composite measures derived from common factor analyses. Therefore, the common factor analysis is not reported in the revised manuscript because there are already individual and linear combinatorial measures that are highly similar to the common factor combination in our analysis. However, if you prefer us to include in the manuscript the results with the factor score, we will include in the next revision, should the manuscript make it to the next round.

Thank you for asking us to test the regression with the factor score.

**Table R1: Alternate dependent variable: *ΔREM2 & ΔREM2_factor***

|  | ***ΔREM2*** | | | | | ***ΔREM2_factor*** | | | |
| --- | --- | --- | --- | --- | --- | --- | --- | --- | --- |
| **Poly. Order** | ***1*** | | ***2*** | | ***3*** | ***1*** | ***2*** | ***3*** | |
| *Unionization* | -0.015 | -0.001 | | -0.028 | | -0.012 | -0.006 | | -0.027 |
|  | (-0.418) | (-0.027) | | (-0.563) | | (-0.720) | (-0.334) | | (-1.270) |
|  | ***ΔREM2_t+1_*** | | | | | ***ΔREM2_factor_t+1_*** | | | |
| *Unionization* | -0.155 | -0.186* | | -0.203* | | -0.086* | -0.108* | | -0.105 |
|  | (-1.567) | (-1.711) | | (-1.755) | | (-1.750) | (-1.955) | | (-1.599) |

3. *Regarding variable measurements, please refer to He et al. (2022) on how to measure the abnormal operating cash flows in a better way.*

The revised manuscript follows He et al. (2022) and constructs an alternative measure of abnormal operating cash flows in robustness tests (read Panel C of Table 3).

*Also, refer to He (2015) to use alternative measures of abnormal accruals for robustness tests.*

He (2015) also used the cross-sectional modified Jones model following Dechow et al. (1995) to proxy for abnormal accruals. The result (read Panel B of Table 10) is consistent with our second discretionary accruals measure. Thank you for helping us test this robustness.

*Regarding the measure of firm risks, please use earnings volatility (in lieu of Altman’s Z score and financial leverage), as the latter two measures reflect more of the needs for cash rather earnings.*

The revised manuscript uses earnings volatility to measure firm risk. Besides, we also include Altman’s Z score as an alternative measure of firm risk. Thank you for the suggestion.

4. *Please maintain the same set of control variables across all the regressions in which the dependent variable is earnings management.*

The revised manuscript uses the same set of control variables for all regressions where earnings management is the dependent variable (read Columns 4-6 of Tables 2, Table 3, and Tables 5-12).

# **Authors’ Responses to Reviewer 2’s Comments**

*Using close-call labor elections data, the authors document that labor unionization has a positive effect on firms’ real earnings management, suggesting the pressure effect of increased labor power. They further find that this effect is attenuated for firms located in right-to-work states, and is strengthened when managers face higher pressure on earnings. Their findings are robust to alternative model specifications and alternative measures of real earnings management. Overall, this manuscript is well written, the logic is clear and the empirical tests are rigorously executed.”*

We greatly appreciate your positive comment on our work.

1. *A central assumption in the manuscript is that when the labor union is powerful, the company is under pressure to manipulate earnings upwards. However, this may not necessarily be the case. Companies also manage earnings downwards to combat labor union demands. For example, when companies have abundant earnings to report, a powerful labor union would request for wage rises for the workers, and the management are reluctant to incur additional labor costs. They would be motivated to manipulate earnings downloads to avert demands from labor unions. The authors could incorporate this possibility in their arguments to offer a more complete picture of how labor unionization affects firms’ accounting choices.*

The coauthor who assembled the sample and analyzed the data was unavailable. Therefore, we assembled a new and updated (until the year 2021 as opposed to 2018) sample and report a new set of empirical results. The new results suggest—consistent with your reasoning—a negative effect of labor unionization on corporate real earnings management, which is consistent with the view that firms will manipulate earnings downwards to combat labor union demands. Accordingly, the revised manuscript cites prior research to provide the reasons why labor unionization can cause managers to adjust earnings manipulation upwards or downwards (read the logic in Section II on pp. 7-8). However, the evidence is in support of the downward management of earnings. Thank you for asking us to check this plausible effect.

2. *The main measures of real earnings management used in this manuscript follow Zang (2012) and Cohen and Zarowin (2010), both are widely used in prior literature. However, Chen, Hribar, and Melessa (2018) show that the two-stage approach of first estimating REM and then using it as a dependent variable causes second-stage coefficients and standard errors to be biased. The authors may refer to this paper for suggestions to overcome these biases.*

We tried to follow Chen, Hribar, and Melessa (2018) to construct the one-stage REM measure. However, their measure of abnormal discretionary expenses (that we name *DISX_one*) does not combine discretionary expenses with abnormal operating cash flows or production costs—the measures that most of the extant research on REM use. As shown in the table below, the effect of EU on *DISX_one* is insignificant in our setting, possibly because the construction of *DISX_one* is inconsistent with the logic of considering DISX in conjunction with production costs or OANCF. Additionally, according to Chen, Hribar, and Melessa (2018), although one-stage measures correct the two-step bias by eliminating the discretion in designating controls as either first- or second-step regressors, the error correction effect may be limited because the one-stage method does not resolve the issues associated with selecting the appropriate set of controls (p. 778 in Chen, Hribar, and Melessa [2018]). Therefore, to avoid errors in calculating one-stage measures (both theoretically and computationally), we do not include this method in the revised manuscript. However, if you prefer us to include this result in the manuscript, we will include it in the next revision, provided the manuscript enters the next round.

**Table R2: Alternate dependent variable: *ΔDISX_one & DISX_one_t+1_***

|  | **Dependent Variable=** | | | | | |
| --- | --- | --- | --- | --- | --- | --- |
|  | ***ΔDISX_one*** | | | ***DISX_one_t+1_*** | | |
| **Poly. Order** | **1** | **2** | **3** | **1** | **2** | **3** |
| *Unionization* | -0.004 | -0.001 | -0.003 | 0.008 | 0.005 | 0.033 |
|  | (-0.162) | (-0.029) | (-0.101) | (0.172) | (0.109) | (0.569) |

3. *In the sample selection part, it is better to include a sample formation procedure (for example, what is the initial sample size, and how the* *data requirements reduce the sample to a final size of the firm-year observations.*

The revised manuscript describes how we assembled the sample and thus explains how/why the sample includes 654 firm-year observations (pp. 9-10).

4. *In the “Measures of Real Earnings Management” part, the sequence of the two equations is mixed. When introducing the measure of abnormal production costs, the equation and related discussions are about DISX. When introducing the measure of abnormal discretionary expenses, the equation and related discussions are about PROD.*

We have solved this problem by rewriting the “Measures of Real Earnings Management” subsection.

5. *In the “HETEROGENEITY TESTS” part, the measures used for right-to-work states, benchmark beating, growth opportunity and corporate risks are not explicitly explained in the main text. At least the authors should briefly introduce the variable names and descriptions for the measures before discuss the empirical results.*

The revised manuscript explicitly states the names of and descriptions of variables used for heterogeneity tests (read Section V, named Heterogeneity Tests on pp. 17-20, the appendix table, and the footers of Tables 5-8).

6. *In the proxies used for external earnings pressure, the “growth opportunity” proxy is not intuitive to me. The authors argue that firms with few growth opportunities are more likely to be financially constrained, but this is not necessarily true. On the contrary, when considering the life cycle of firms, firms at their maturity stage have less growth potentials, but their operating cash flows just get to peak. Therefore, growth opportunity is a noisy proxy for external earnings pressure.*

We dropped the growth opportunity proxy because it is a noisy measure of external earnings pressure. Instead, the revised manuscript uses leverage and cash flows as alternate measures of firm pressure. Because firms with less financial constraints have more space to manipulate earnings downwards to combat labor union demands, the negative effect of labor unionization on real earnings management is more pronounced for firms with lower leverage or higher cash-flow level.

7. *In the subsample analysis for Tables 4-7, for several columns, the coefficients on Unionization are both significant in two groups, and only the magnitudes of the coefficients diff. Is there a way to test the statistical difference in the coefficients in the subsamples? My concern is that there is actually no significant statistical difference in the coefficients of Unionization in the two groups.*

The new subsample analyses test the statistical difference in the coefficients. We find significant differences in the coefficients of *Unionization* in the two groups (read Tables 5-8).

8. *In Table 11, when discussing the economic significance of the coefficients, it’s more informative to illustrate how a standard deviation change in X leads to a certain standard deviation change in Y.*

The revised manuscript (p. 23, Table 12) reports the economic significance of the coefficients in terms of how much change in Y is caused by one-standard deviation change in X. Thank you for your suggestion!

9. *Some typos found in the main text: Page 7: The numbers follow Section are missing ?Page 8: Although wining the labor election increases labor power”*

We have fixed these errors in the revised manuscript. Sorry about them.

# **References**

Chen, W. E. I., Hribar, P., & Melessa, S. (2018). Incorrect inferences when using residuals as dependent variables. *Journal of Accounting Research*, 56(3), 751-796.

Cheng Q, Lee J, Shevlin T J. (2016) Internal Governance and Real Earnings Management. *Accounting Review*, 91(4): 1051-1085.

He, G. (2015). The effect of CEO inside debt holdings on financial reporting quality. *Review of Accounting Studies*, 20, 501-536.

He, G., Li, A. Z., & Shen, D. (2021). The role of earnings management in equity valuation. *Encyclopedia of Finance*. Cham: Springer International Publishing, 2021. 1-35.
