# Supplementary material for: Labor unionization and real earnings management: Evidence from labor elections
Source: PLoS One. 2024 Feb 20;19(2):e0292889. doi: 10.1371/journal.pone.0292889 (PMC10878506; doi:10.1371/journal.pone.0292889)
Supplement: Attachment — Submitted filename: Response to reviewers.docx [file pone.0292889.s006.docx]

# **Labor Unionization and Real Earnings Management: Evidence from Labor Elections**

# **Authors’ Responses to the Reviewer Comments on PONE-D-23-09561.R1**

We thank the editor and the two reviewers for their feedback, which strengthened our manuscript. We reproduce the reviewer’s comments in italics and our response in regular font.

# **Authors’ Responses to Reviewer 1’s Comments**

*I see quite a few merits and strengths with the revised paper. Well done! I knew there are other papers (e.g., Chang et al. 2022, Journal of Corporate Finance), which examine the same research question as does this paper under review. However, I realize there are substantial differences between the competing papers and the authors' paper. In my opinion, so long as the topic is important, it warrants several papers shedding light on it from different perspectives and based on different research designs/samples. I suggest that the authors just use a footnote to tell the differences between their paper and the competing papers*.

We thank you for finding merit and strengths in our revised manuscript. Following your suggestion, footnote 9 on page #13 of the revised manuscript states how our manuscript differs from published research articles on the topic of labor unionization and real earnings management.

*That done, the authors need to proof-read the paper carefully, and also make the citations consistent between the text and the reference list. Thank you for the opportunity to read this paper. It is a very good one for publication.*

We have also checked the consistency between citations and references. Specifically, we have added Chang et al. (2022) to the reference list and dropped Farrell and Whidbee (2003), Francis et al. (2008), Matsunaga and Park (2001), and Opler and Titman (1994) which are no longer related to the revised manuscript. Thank you for asking us to check.

# **Authors’ Responses to Reviewer 2’s Comments**

*The authors have well addressed all my comments in the previous round. My only concern is, in this version, the authors have extended the sample period from 2018 to 2022, but now the results have been opposite. Therefore, I'm a bit concerned about the robustness of the results. The authors need to provide more justifications for the data in this version. Is the opposite result driven by intertemporal changes, or restructure of data in all periods?*

Thank you for asking. We believe the opposite result is mainly due to intertemporal changes. On the one hand, the intertemporal changes in the sample may play a role in the variation in the regression results, where some earliest labor union election cases (before 1988, when the NLRB database was not complete) are replaced with the most recent cases (after 2018). On the other hand, the REM measure depends on the calculation of *REM_OANCF*, *REM_DISX,* and *REM_DISX*, which are residuals obtained from different equations (see the “Measures of REM” part on pages #11-13). And regressions based on the renewed sample may yield different residuals, resulting in changes in REM, as well as our main empirical results. Besides, although we create a new data set and report a new set of empirical results due to the previous data being unavailable, we strictly follow the sample construction procedure recorded in the previous manuscript and our variables are comparable to the previous ones in terms of sample size and values. Therefore, we think the opposite result is probably driven by intertemporal changes, rather than the restructuring of data in all periods.
