# Supplementary material for: Labor unionization and real earnings management: Evidence from labor elections
Source: PLoS One. 2024 Feb 20;19(2):e0292889. doi: 10.1371/journal.pone.0292889 (PMC10878506; doi:10.1371/journal.pone.0292889)
Supplement: Attachment — Submitted filename: Referee report for PONE-D-23-07458.pdf [file pone.0292889.s004.pdf]

Referee report on "Labor Unionization and Real Earnings Management: Evidence from Labor Elections"

Summary

Using close-call labor elections data, the authors document that labor unionization has a positive effect on firms' real earnings management, suggesting the pressure effect of increased labor power. They further find that this effect is attenuated for firms located in right-to-work states, and is strengthened when managers face higher pressure on earnings. Their findings are robust to alternative model specifications and alternative measures of real earnings management.

Comments

In overall, this manuscript is well written, the logic is clear and the empirical tests are rigorously executed. My specific comments on the paper are stated below.

1. A central assumption in the manuscript is that when the labor union is powerful, the company is under pressure to manipulate earnings upwards. However, this may not necessarily be the case. Companies also manage earnings downwards to combat labor union demands. For example, when companies have abundant earnings to report, a powerful labor union would request for wage rises for the workers, and the management are reluctant to incur additional labor costs. They would be motivated to manipulate earnings downloads to avert demands from labor unions. The authors could incorporate this possibility in their arguments to offer a more complete picture of how labor unionization affects firms' accounting choices.

2. The main measures of real earnings management used in this manuscript follow Zang (2012) and Cohen and Zarowin (2010), both are widely used in prior literature. However, Chen, Hribar, and Melessa (2018) show that the two-stage approach of first estimating REM and then using it as a dependent variable causes second-stage coefficients and standard errors to be biased. The authors may refer to this paper for suggestions to overcome these biases.

3. In the sample selection part, it is better to include a sample formation procedure (for example, what is the initial sample size, and how the data requirements reduce the sample to a final size of 562 firm-year observations).

4. In the "Measures of Real Earnings Management" part, the sequence of the two equations is mixed. When introducing the measure of abnormal production costs, the equation and related discussions are about DISX. When introducing the measure of abnormal discretionary expenses, the equation and related discussions are about PROD.

5. In the "HETEROGENEITY TESTS" part, the measures used for **right-to-work states, benchmark beating, growth opportunity and corporate risks** are not explicitly explained in the main text. At least the authors should briefly introduce the variable names and descriptions for the measures before discuss the empirical results.

6. In the proxies used for external earnings pressure, the "growth opportunity" proxy is not intuitive to me. The authors argue that firms with few growth opportunities are more likely to be financially constrained, but this is not necessarily true. On the contrary, when considering the life cycle of firms, firms at their maturity stage have less growth potentials, but their operating cash flows just get to peak. Therefore, growth opportunity is a noisy proxy for external earnings pressure.

7. In the subsample analysis for Tables 4-7, for several columns, the coefficients on Unionization are both significant in two groups, and only the magnitudes of the coefficients diff. Is there a way to test the statistical difference in the coefficients in the subsamples? My concern is that there is actually no significant statistical difference in the coefficients of Unionization in the two groups.

8. In Table 11, when discussing the economic significance of the coefficients, it's more informative to illustrate how a standard deviation change in X leads to a certain standard deviation change in Y.

9. Some typos found in the main text:

Page 7: The numbers follow Section are missing?

The remainder of the paper proceeds as follows. Section □ reviews related literature and develops our hypotheses. Section □ describes sample and methodology. Section □ presents our main empirical results. Section □ shows results of heterogeneity tests. Section □ provides addition tests. Section □ concludes.

Page 8: Although ***wining*** the labor election increases labor power
